# Sex differences in body composition affect total airway resistance during puberty

**DOI:** 10.1186/s12887-022-03198-1

**Published:** 2022-03-17

**Authors:** Ju Hee Kim, Jin Ah Kim, Eun Kyo Ha, Hye Mi Jee, Seung Won Lee, Mo Kyung Jung, Sanghoo Lee, Yoon Ho Shin, Eun-Gyong Yoo, Man Yong Han

**Affiliations:** 1 Department of Pediatrics, Kangdong Sacred Heart Hospital, Hallym University Medical Center, Seoul, Korea; 2 Department of Pediatrics, Seoul National University Hospital, Seoul National University College of Medicine, Seoul, Korea; 3 Department of Pediatrics, Kangwon National University Hospital, Gangwon-do, Korea; 4 Department of Pediatrics, Kangnam Sacred Heart Hospital, Hallym University Medical Center, Seoul, Korea; 5 Department of Pediatrics, CHA Bundang Medical Center, CHA University School of Medicine, Seongnam, Korea; 6 Department of Data Science, Sejong University College of Software Convergence, Seoul, Korea; 7 Sungkyunkwan University School of Medicine, Suwon, Korea; 8 Center for Companion Biomarker, Seoul Clinical Laboratories Healthcare, Yongin, Korea; 9 Department of Pediatrics, CHA Gangnam Medical Center, CHA University School of Medicine, Seoul, Korea

## Abstract

**Background:**

During puberty, changes in body composition due to sex hormones are associated with lung mechanics. However, little is known about the mediation effect of sex differences in body composition during puberty with total airway resistance.

**Methods:**

We prospectively recruited 620 children (10–12 years old) from the general population and conducted a cross-sectional study. This study assessed pubertal status according to the five Tanner stages using a questionnaire, line drawings, and each subject’s blood sex hormone profile. Both the impulse oscillation system for total lung mechanics and multifrequency bioelectrical impedance for body composition analyses were conducted. The effects of puberty on body composition and subsequent total lung resistance were evaluated using mediation analysis.

**Results:**

Among the 503 children enrolled, there were 261 males (51.9%) and 242 females (48.1%). In males, higher testosterone levels corresponded with reduced total lung resistance (β = –0.13, 95% CI = –0.21 to –0.05, *p* < 0.001), and the proportion of the mediating effect through the muscle-fat ratio was 19% (95% CI = 4 to 59, *p* = 0.02). In contrast, in females, pubertal status reduced total lung resistance (β = –0.27, 95% CI = –0.58 to –0.05, *p* = 0.04), however, the proportion of the mediating effect through the body mass index was –51% (95% CI = –244 to –4%, *p* = 0.04).

**Conclusion:**

The muscle-fat ratio in adolescent males had a synergistic effect with testosterone on improving total airway resistance, whereas improvements in lung resistance by pubertal status were partially masked by body mass index in adolescent females. In conclusion, body composition changes during puberty between males and females have differing effects on total airway resistance.

## Introduction

Although males have a larger lung size than females, the lungs of females tend to have lower airway resistance and higher airway conductance in young childhood [1]. In addition, females have higher threshold responses to inhaled methacholine and a decreased prevalence of airway hyper-responsiveness throughout childhood. However, these findings were reversed in males and females after puberty [1, 2]. Several studies attributed these sex differences to the immunologic functions of sex hormones or changes in lung physiology related to sex and age, but the underlying mechanisms remain elusive [3].

Sex differences in body composition become striking with the increased release of sex hormones during puberty, such as increased fat gain by adolescent females compared to increased fat-free skeletal mass gain by adolescent males during this period [4]. Moreover, body composition, including body mass index (BMI), fat mass, and muscle mass, was significantly associated with pulmonary function in males and females [5–8]. Despite these previous findings, little is known about the different mediating effects of body composition changes between males and females on the association between puberty and total airway resistance. Therefore, we aimed to investigate the different mediating effects of sex-dependent changes in body composition on total airway resistance during puberty.

## Methods

### Study population

This general population-based cross-sectional study examined children (aged 10 – 12 years) from 11 elementary schools as part of the Seongnam Atopy Project in Korea [9]. Questionnaires were administered to the parents of 2,382 children, and 745 completed questionnaires were collected. A total of 125 children were excluded due to the absence of written consent or incorrect questionnaires; thus, 620 children were eligible. Questionnaires were used to obtain the demographic and clinical variables of all participants. Of the 620 children, 503 children who underwent impulse oscillometry (IOS), their blood and body composition tests were included. The study protocol was approved by the Institutional Review Board of CHA University, and written informed consent was obtained from the parents or caregivers (2017–04-049).

### Impulse oscillometry (IOS)

IOS was performed based on current guidelines [10]. Airway resistance at 5 Hz (Rrs5) was measured using a Jaeger MasterScreen IOS system (Jaeger Company, Wurzburg, Germany). The signal duration was 30 s or longer, and measurements of three acceptable maneuvers were acquired at each time while monitoring the flow curves. Rrs5 is the total airway resistance [11].

### Laboratory tests

Venous serum was obtained from participants at their elementary schools. All serum samples were safely transported to the Department of Laboratory Medicine at Bundang CHA hospital by putting them in a dedicated transport box that is kept refrigerated at 2 to 4℃. Thereafter, all serum sample were centrifuged within 2 h and stored at –80 ºC until the day of the study. Blood testosterone levels were determined using a Cobas 8000 c702 Chemistry Autoanalyzer (Roche, Basel, Switzerland). Levels of 25-OH vitamin D were measured using a fluorescent enzyme-linked immunosorbent assay kit (Immunodiagnostic Systems, COBAS 6000 Roche, Mannheim, Germany). Six types of serum inhalant allergen-specific IgE (Alternaria, birch, cat dander, dog dander, Dermatophagoides farina, Japanese hop) and total IgE levels were measured using the ImmunoCAP system (Phadia AB, Uppsala, Sweden). Allergen-specific serum IgE levels ≥ 0.35 kU/L were defined as positive for each allergen.

### Pubertal development

Assessments of pubertal status in females were based on the development of breasts using the Tanner stages and a detailed questionnaire with line drawings [12]. Breast development is divided into five stages: 1 (preadolescent), 2 (breast buds), 3 (areola darkens and breast tissue enlarges), 4 (areola and nipple ridge), and 5 (nipple protrusion and fully developed breast) [13]. Female pubertal status was dichotomized into early (1 and 2) and late (3, 4, and 5) puberty stages [14]. Pubertal status in males was assessed using log-substituted testosterone levels [15].

### Body composition

Multifrequency bioelectrical impedance (InBody 720; Biospace, Tokyo, Japan) was used to measure body composition. Children participating in the bioelectrical impedance analysis adhered to the following protocol: fasting for at least three hours, empty bladder just before bioelectrical impedance analysis, and avoidance of vigorous physical activity. Parameters including height, weight, BMI, and muscle-fat ratio were automatically measured within 2 min. BMI is used as an indirect indicator of body fat mass in children due to its high correlation with body fat mass [16, 17]. The muscle-fat ratio has been used to assess the association between muscle and fat mass [18].

### Statistical analysis

The relationship between pubertal status (testosterone level and Tanner stage) and body composition (muscle-fat ratio and BMI), body composition and Rrs5, and pubertal status and Rrs5 were determined using generalized linear regression with a gamma function, with adjustment for confounding factors (prematurity [< 37 weeks’ gestation], low birth weight [< 2500 g], passive smoking, aeroallergen sensitization, history of wheezing episode, and 25-OH vitamin D levels). Analyses were performed using SPSS (version 24.0; IBM Corp., Armonk, NY, USA). The “Mediation” package from R (version 3.1.0) was used to quantitatively estimate the mediating and direct effects of body composition factors on the association between pubertal status and pulmonary function, with adjustment for confounding factors. This package estimated confidence intervals (CIs) using bootstrapping with 1000 resamples. Statistical significance was defined as a two-sided *p* value < 0.05.

## Results

### Population characteristics

The demographic and clinical characteristics of participants are presented in Table 1. There were 261 males (51.9%) and 242 females (48.1%). The mean values of height and weight were 149.61 cm (standard deviation [SD], 7.99 cm) and 43.11 kg (SD, 9.57 kg) in males and 149.53 cm (SD, 7.48 cm) and 42.47 kg (SD, 8.77 kg) in females, respectively, and there were no significant differences between sexes. Birth weight was higher in males (mean [SD], 3.30 kg [0.43 kg]) than in females (mean [SD], 3.16 kg [0.43 kg]). Allergen sensitization was more prevalent in males (168, 73.0%) than in females (131, 57.7%). Furthermore, Rrs5 was higher in males (median [interquartile range {IQR}], 4.99 hPa/L/s [4.42 to 5.77 hPa/L/s]) than in females (4.79 hPa/L/s [4.21 to 5.46 hPa/L/s]). There was a greater number of females in late pubertal stage (143, 61.1%) than males (29, 12.7%).

**Table 1:** Demographic and clinical characteristics of study subjects (*n* = 503)

	Male (*n* = 261, 51.9%)	Female (*n* = 242, 48.1%)
Anthropometrics		
Age, years, mean (SD)	11.03 (0.70)	11.05 (1.03)
Height, cm, mean (SD)	149.61 (7.99)	149.53 (7.48)
Weight, kg, mean (SD)	43.11 (9.57)	42.47 (8.77)
Birth characteristics		
Gestational age, weeks (SD)	38.77 (2.15)	38.98 (2.06)
Birth weight, kg, mean (SD)	**3.30 (0.43)**	**3.16 (0.43)**
Passive smoking, n (%)	100 (43.5)	103 (45.4)
Aeroallergen sensitization^a^, n (%)	**168 (73.0)**	**131 (57.7)**
Wheeze episodes, n (%)		
None	225 (86.2)	219 (90.9)
1 or over	36 (13.8)	22 (9.1)
25-OH vitamin D, ng/mL, GM (GSD)	**22.16 (1.28)**	**19.15 (1.33)**
Total IgE, IU/mL, GM (GSD)	118.30 (3.53)	94.91 (3.60)
Oscillometric lung function, median (IQR)		
Rrs5, hPa/L/s	**4.99 (4.42 to 5.77)**	**4.79 (4.21 to 5.46)**
Body composition		
BMI z score, mean (SD)	− 0.08 (1.04)	0.04 (1.05)
Muscle fat ratio, median (IQR)	3.54 (2.44 to 5.53)	2.93 (2.16 to 3.91)
Pubertal status, n (%)		
Early pubertal stage^b^	**200 (87.3)**	**91 (38.9)**
Late pubertal stage^b^	**29 (12.7)**	**143 (61.1)**
Testosterone, ng/mL, median (IQR)	**0.70 (0.12 to 2.49)**	**0.19 (0.12 to 0.27)**

*Abbreviations*: *n* number, *SD* Standard deviation, *GM* Geometric mean, *GSD* Geometric standard deviation, *IQR* Inter quartile range, *IgE* Immunoglobulin E, *Rrs5* Resistance at 5 Hz, *BMI* Body mass index

^a^Aeroallergen sensitization was defined as an IgE value of 0.35 kU/L or more in response to at least 1 of 6 allergens (*Dermatophagoides farina,* Alternaria, birch, Japanese hop, cat dander, and dog dander)

^b^Early and late pubertal status were determined by the breast development in females and the genital development in males of Tanner stage

Missing data, Birth characteristics, *n* = 8; Passive smoking, *n* = 3; Aeroallergen sensitization, Total IgE, and 25-OH vitamin D, *n* = 33; Wheeze episodes, *n* = 1; Pubertal status, *n* = 40

Numbers in bold indicate significant differences (*P* < 0.05)

### Association between pubertal status, body composition, and lung function

**Fig. 1:**
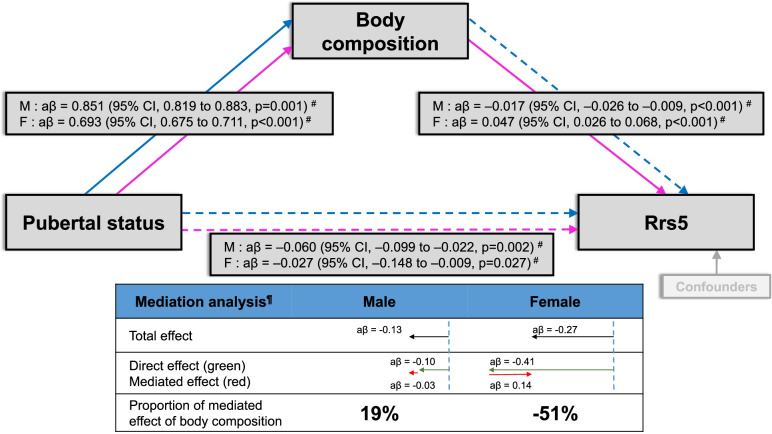
Differences in body composition changes in males (M) and females (F) during puberty have different mediating effects on total airway resistance (Rrs5). Pubertal status refers to logarithmic testosterone levels in males and breast development by Tanner stage in females. Body composition is based on the muscle-fat ratio in males and body mass index (BMI) in females. The blue and pink colored lines represent males and females, respectively. The solid line indicates a positive relationship, whereas a dotted line indicates a negative relationship. Higher testosterone levels in males correlated with a higher muscle-fat ratio, which had a synergic mediating effect (aβ =  − 0.03, proportion = 19%) on lowering airway resistance. Conversely, a higher BMI in females had a negative effect (aβ = 0.14, proportion =  − 51%) on airway resistance. ^#^Generalized linear regression with gamma function adjusted for confounding factors. ^¶^ Mediation package with adjustment for confounding factors. Confounding factors included height, prematurity (< 37 weeks’ gestation), low birth weight (< 2500 g), passive smoking, aeroallergen sensitization, history of wheezing episode, and 25-OH vitamin D levels. Rrs5, resistance at 5 Hz; CI, confidence interval

Figure 1 shows all associations between pubertal status and Rrs5, pubertal status and body composition, and body composition and Rrs5 in males and females. First, pubertal status was negatively associated with the numerical value of Rrs5 in males and females (β [95% CI], –0.06 [–0.10 to –0.02] and –0.03 [–0.15 to –0.01], respectively). Secondly, pubertal status was positively associated with the muscle-fat ratio in males and BMI in females (β [95% CI], 0.85 [0.82 to 0.88] and 0.69 [0.68 to 0.71], respectively). Thirdly, a high muscle-fat ratio correlated with decreased Rrs5 in males (β [95% CI], –0.02 [–0.03 to –0.01]), while a high BMI correlated with increased Rrs5 in females (β [95% CI], 0.05 [0.03 to 0.07]).

Causal mediation analysis indicated that the total effect of pubertal status on Rrs5 in males (β =  − 0.13 [95% CI =  − 0.21 to − 0.05], *p *< 0.001) was due to a direct effect (β =  − 0.10 [95% CI, − 0.20, − 0.03], *p* < 0.001), which was complemented by the mediating effect of the muscle-fat-ratio (β =  − 0.03 [95% CI =  − 0.05 to − 0.01], *p* < 0.001, portion of mediated effect = 19% [95% CI = 4% to 59%], *p* = 0.02). In contrast, the total effect of pubertal status on Rrs5 in females (β =  − 0.27 [95% CI =  − 0.58 to − 0.05], *p* = 0.04) was due to a direct effect (β =  − 0.41 [95% CI, − 0.70 to − 0.17], *p* < 0.001), which was partially offset by the mediating effect of BMI (β = 0.14 [95% CI = 0.03 to 0.28], portion of mediated effect =  − 51% [95% CI =  − 244 to − 4%], *p* = 0.04).

## Discussion

Our study showed that pubertal children have sex-dependent differences in body composition due to the effects of sex hormones, which in turn affect lung function. This may explain the superior lung function of girls before puberty and that of boys after puberty. Increased testosterone levels in boys increases the muscle-fat ratio, which had a synergistic mediating effect on lowering lung resistance. Conversely, the effect of increased BMI upon sexual maturity in females had a negative effect on pulmonary function.

Thus, our study demonstrates that changes in body composition help explain the association between pubertal status and airway resistance. Specifically, the release of sex hormones increases during puberty, and there are sex-dependent immunologic differences in sex hormones. Testosterone has an immunosuppressant effect that reduces systemic and airway inflammation [19], whereas estradiol affects immune cells by promoting Th2 polarization, switching of B cells to IgE production, and degranulation of mast cells and basophils [20, 21]. In addition, puberty in adolescent males and females has different effects on body composition [4], which is consistent with our results. Increased testosterone levels in males promotes myogenic differentiation of multipotent mesenchymal stem cells, stimulates muscle protein synthesis, and inhibits proteolysis [22]. In contrast, the endocrine effects of estradiol promote fat mass accumulation predominantly in the abdomen or hip of females [23]. A proposed mechanism by which body composition could affect lung function is that high lean body mass leads to increased mechanical power of breathing and better resultant lung function, whereas high fat mass induces inflammation of lung tissue, causing a reduction in airway diameter [7].

Several studies have discussed connections between some of these factors, but the present study is the first to identify the links between all three factors. The presence of the links described here is easily understood and straightforward; however, to the best of our knowledge, this study is the first to verify the effect of body composition on changes in pulmonary function during puberty in adolescents from the general population.

We utilized the Rrs5 parameter of the IOS to evaluate total airway resistance. IOS requires only passive and minimal cooperation [24], thus, it is a reliable and noninvasive method to assess airway resistance in children [25]. Several studies have confirmed the usefulness of IOS in diagnosing and monitoring patients with asthma, particularly in children [26]. Although spirometry is the gold standard for the diagnosis of asthma, the Rrs5 parameter of IOS may be a useful parameter to estimate the association of pubertal status and body composition with airway resistance in children.

This study has several limitations. First, this was not a longitudinal study, so causal inferences regarding the relationship between body composition, pubertal status, and lung function cannot be made. Therefore, further studies with prospective follow-up are necessary to confirm our results. Second, there was a lack of information about demographic characteristics, including socioeconomic status and eating or exercise habits, which can affect body composition or airway resistance. However, we tried to analyze the associations between several confounding factors (prematurity, birth weight, passive smoking, allergen sensitization, history of wheezing episode, and 25-OH vitamin D levels). Third, the Tanner stage of each child was determined from a questionnaire answered by the parents rather than a physical examination by a physician. However, self-assessment was considered sufficient for our study because we only distinguished between pre-puberty and puberty [12].

Our study provides new insights into the mediating effect of sex-dependent body composition changes on airway resistance during puberty. Our study will help to guide further research that examines the effects of altering body composition on pulmonary function in children, especially in adolescents with reduced lung function during puberty.

## Data Availability

The datasets used during the current study are available from the corresponding author upon reasonable request.
